# Epigenetic dysregulation of *TET2* in human glioblastoma

**DOI:** 10.18632/oncotarget.25406

**Published:** 2018-05-25

**Authors:** María G. García, Antonella Carella, Rocío G. Urdinguio, Gustavo F. Bayón, Virginia Lopez, Juan Ramón Tejedor, Marta I. Sierra, Estela García-Toraño, Pablo Santamarina, Raúl F. Perez, Cristina Mangas, Aurora Astudillo, M. Daniela Corte-Torres, Inés Sáenz-de-Santa-María, María-Dolores Chiara, Agustín F. Fernández, Mario F. Fraga

**Affiliations:** ^1^ Institute of Oncology of Asturias (IUOPA), HUCA, Universidad de Oviedo, Oviedo, Spain; ^2^ Nanomaterials and Nanotechnology Research Center (CINN-CSIC), Universidad de Oviedo, El Entrego, Asturias, Spain; ^3^ Fundación para la Investigación Biosanitaria de Asturias (FINBA), Instituto de Investigación Sanitaria del Principado de Asturias (ISPA), Oviedo, Asturias, Spain; ^4^ Department of Pathology, Hospital Universitario Central de Asturias (HUCA), Oviedo, Spain; ^5^ Biobanco del Principado de Asturias, Hospital Universitario Central de Asturias (HUCA), Oviedo, Spain; ^6^ Otorhinolaryngology Service, Hospital Universitario Central de Asturias, Instituto Universitario de Oncología del Principado de Asturias, Universidad de Oviedo, CIBERONC, Oviedo, Spain

**Keywords:** 5-hydroxymehtylcytosine, glioblastoma, TET2, epigenetics, DNA methylation

## Abstract

Ten-eleven translocation (TET) enzymes are frequently deregulated in cancer, but the underlying molecular mechanisms are still poorly understood. Here we report that *TET2* shows frequent epigenetic alterations in human glioblastoma including DNA hypermethylation and hypo-hydroxymethylation, as well as loss of histone acetylation. Ectopic overexpression of *TET2* regulated neural differentiation in glioblastoma cell lines and impaired tumor growth. Our results suggest that epigenetic dysregulation of *TET2* plays a role in human glioblastoma.

## INTRODUCTION

The methylcytosine dioxygenase TET2 is a member of the TET (ten-eleven translocation) gene family. Initially, this family of enzymes was proposed to be involved in DNA demethylation in mammals because they are able to catalyze the conversion of 5-methylcitosine (5 mC) in 5-hydroxymethylcitosine (5 hmC) [1]. This is an oxidative reaction that requires 2-oxoglutarate- (2-OG) and Fe(II) as cofactors. All TET proteins can further oxidize 5 hmC to 5-formylcytosine (5fC) and 5-carboxylcytosine (5caC) [2]. In addition to its possible role in active DNA demethylation, 5 hmC has also recently been proposed as an epigenetic mark in its own right, and having a functional role in chromatin regulation and epigenetic maintenance [3]. TET protein activity and 5 hmC profiles are involved in many biological processes such as, among others, gene transcription, DNA regulation, tumorigenesis and cell function, development and differentiation.

The recruitment of TET2 to DNA and its activity is regulated by several factors. TET2 DNA binding sites are associated with IDAX, and is also related to TET2 repression [4]. It is also known that this enzyme can be externally modulated by other genes, such as Wilms' tumor 1 gene (WT1) [5, 6], elements such as the microRNA miR-22 [7], and, in a site-specific manner, it is mediated by transcription factors such as EBF1 (early B-cell factor 1) [8] among others.

TET2 activity is involved in many of the biological processes, including the embryogenesis. During zygote formation, expression of TET2 is low, but levels increase in the blastocyst stage and during germ layer differentiation. Alterations in embryonic development and cell differentiation process [9, 10], especially in hematopoietic lineages [11, 12], have been identified in different TET2 deficient mice models. TET2 expression levels are tissue dependent, and the consistent expression found mainly in hematopoietic [13, 14] and neural lineages [15–17] in adults, highlights the special importance that TET2 probably plays in these tissues.

TETs are dysregulated in a number of pathological processes. Aberrant 5 hmC patterns are common in certain tumor types [18, 19], and TET2 has been described as a tumor suppressor gene in many cancers [20, 21]. TET2 downregulation has been studied in a large range of hematological malignances, with various mutations in the gene having been indicated as the principal cause [13, 22, 23]. However, in other cases, TET2 activity decreases as result of mutations in or disorders of other enzymes such as isocitrate dehydrogenase (IDH) [24]. TET2 dysregulation is also present in other tumor types [25–27], in some of which TET2 downregulation has been found to be associated with poor prognosis and reduced patient survival [28–30].

Despite the large numbers of studies carried out the mechanisms that generate this loss of TET2 protein in some tumors is not yet clearly understood. Here we show that epigenetic mechanisms might play an important role in the aberrant regulation of TET2 in human glioma.

## RESULTS

### Frequent epigenetic dysregulation of TET2 hydroxymethylation in human glioblastoma

**Figure 1:**
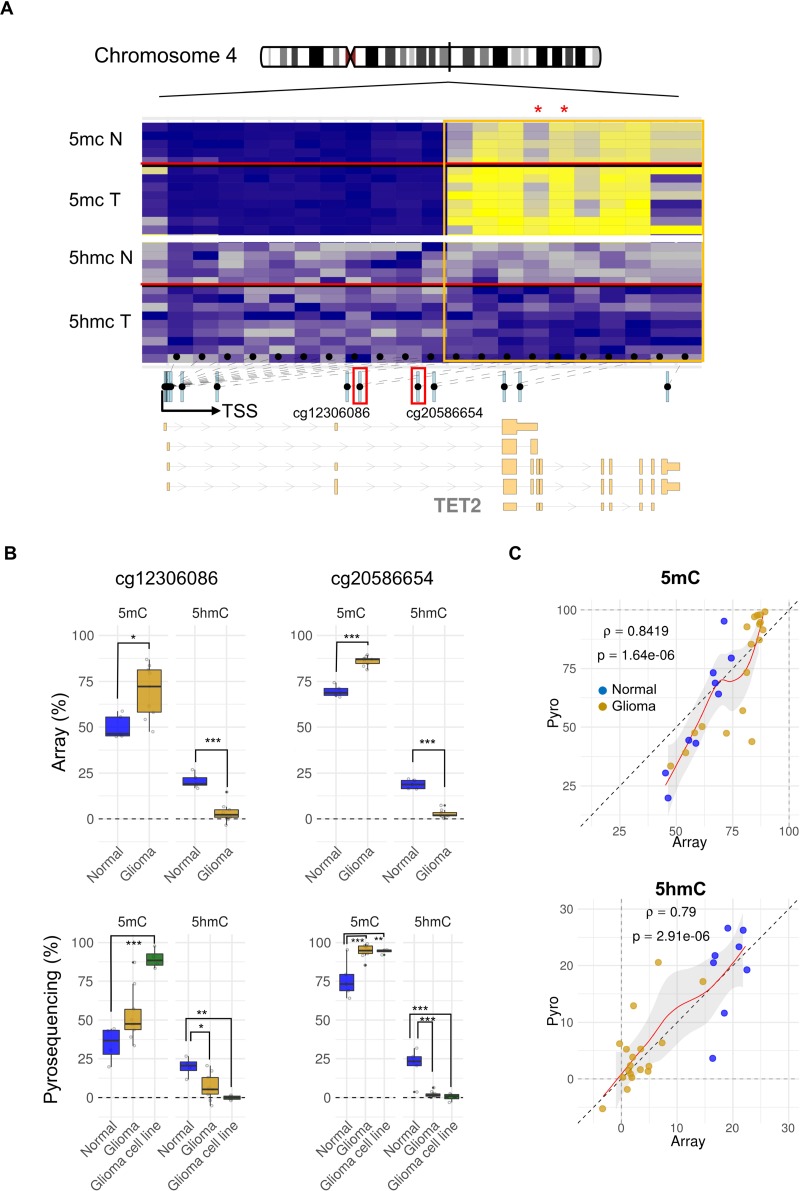
Locus specific TET2 alterations of DNA methylation and hydroxymethylation in glioma (**A**) Schematic representation of gene organization. Exons, introns, and transcription start site (TSS) locations are shown below. The 21 CpG positions along TET2 analyzed in the Illumina methylation array are indicated by black dots (intragenic CpG sites are bounded by the orange frame). Locus-specific patterns of DNA methylation (blue to yellow) and hydroxymethylation (gray to blue) in 5 non-tumorigenic human brain and 9 glioblastoma samples are shown. Location of representative CpGs selected for study (cg12306086 and cg20586654) are indicated by the small red boxes below the heatmap, in which they are marked with asterisks. (**B**) Box plot showing differences between the average percentage of 5 mC and 5 hmC in both normal brain and glioma samples of the two-representative intragenic CpGs studied (cg12306086 and cg20586654) (upper part). Technical validation by bisulfite pyrosequencing of 5 mC and 5 hmC changes occurring among normal samples (*n* = 5), primary tumors (*n* = 9) and glioblastoma cell lines (*n* = 4) are shown below. *p*-values were adjusted by applying the Bonferroni correction. ^*^p-value < 0.05; ^**^p-value < 0.01. (**C**) Scatter plots showing the percentage of 5 mC (upper panel) and 5 hmC (lower panel) in normal (blue plots) and tumoral (yellow plots) samples obtained by pyrosequencing (y-axis) and arrays (x-axis). ρ: Spearman rank correlation.

To study the possible aberrant epigenetic regulation of TET2 in brain tumors, we first used data from the 450K Infinium Illumina methylation platform. We determined the DNA methylation and hydroxymethylation status of 21 CpG positions within TET2 promoter DNA regions and the gene body in 9 samples obtained from patients with glioblastoma and 5 from non-tumorigenic brain samples. This revealed a locus-specific pattern of DNA methylation and hydroxymethylation alterations in tumoral samples (Figure 1A). CpG sites located at the promoter CpG island presented very low levels of 5 mC and of 5 hmC in both brain and glioblastoma samples. In contrast, intragenic CpG sites presented much higher 5 hmC levels in the non-tumorigenic samples compared with those from tumors (on average, 15% and 2%, respectively), while intragenic 5 mC levels were higher (10%) in tumoral samples.

To validate the data obtained with the methylation arrays we used bisulfite pyrosequencing to analyze the DNA methylation and hydroxymethylation status of two CpG sites located at intron 2 of TET2 (cg12306086 and cg20586654) in the same samples analyzed in the methylation arrays, as well as in additional glioblastoma cell lines (see material and methods). The results confirmed the 5 mC and 5 hmC profiles obtained in the arrays (Figure 1B–1C). Moreover, these experiments revealed a similar pattern of 5 mC and 5 hmC at two neighboring CpG sites of the cg12306086 position (Supplementary Figure 1A). The loss of 5 hmC in glioblastoma was further validated by the results of an alternative technique which is not dependent on the oxidative bisulfite conversion and based instead on DNA immunoprecipitation with an antibody against 5 hmC (Supplementary Figure 2).

Next, we used bisulfite pyrosequencing to analyze the 5 mC and 5 hmC levels at the cg20586654 CpG position in an independent cohort of 8 glioblastomas and 7 non-tumorigenic brains. We detected TET2 hypermethylation and hypo-hydroxymethylation in all the tumoral samples analyzed (Supplementary Figure 1B), which confirmed that this is a frequent event both *in vivo* and *in vitro*.

To identify other possible epigenetic mechanisms involved in TET2 regulation, we carried out quantitative ChIP analyses on brain and glioma samples using an antibody against H4K16 acetylation, a histone post-translational modification associated with gene activation [31]. We found a decrease in H4K16ac in the tumoral sample (Supplementary Figure 3), further supporting the idea that, in addition to DNA methylation and DNA hydroxymethylation, histone post-translational modifications could have an important role in TET2 regulation in glioma.

### Epigenetic dysregulation is associated with TET2 repression in glioblastoma

**Figure 2:**
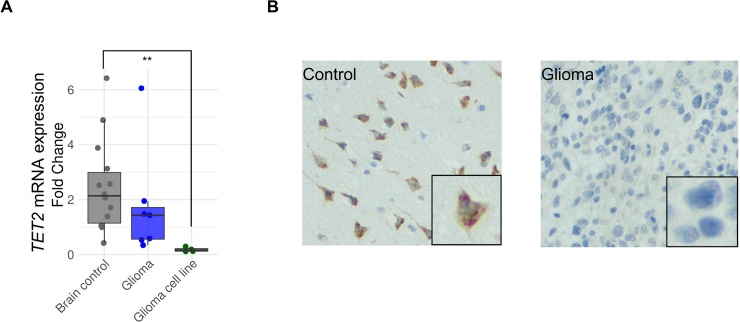
TET2 expression is downregulated in glioma (**A**) Comparison of TET2 mRNA expression levels between control brains (*n* = 14), gliomas (*n* = 7) and glioblastoma cell lines (*n* = 4) are shown on the left. Expression levels were determined by qRT-PCR, and data are expressed as a ratio in relation to GAPDH. Fold change was calculated relative to control samples with low expression levels. Wilcoxon signed rank test was applied. ^**^p-value < 0.01. (**B**) TET2 protein levels detected by immunohistochemistry (IHC) are shown.

To determine the possible role of TET2 epigenetic dysregulation in gene expression, we first compared the mRNA expression level of TET2 in 14 non-tumorigenic brain samples, 7 samples obtained from patients with glioblastoma multiforme and 4 glioblastoma cell lines. TET2 expression was lower in primary glioblastomas and glioblastoma cell lines than in control samples (Wilcoxon rank sum test, *p*-value < 0.05 for cell lines) (Figure 2A). However, in all the glioblastoma cancer lines analyzed, TET2 expression was only just above detectable levels (Figure 2A). In the same vein, TET2 protein expression was stronger in the control brain samples than in primary glioblastoma samples (Figure 2B).

### Ectopic overexpression of TET2 reduces cell growth *in vitro*

**Figure 3:**
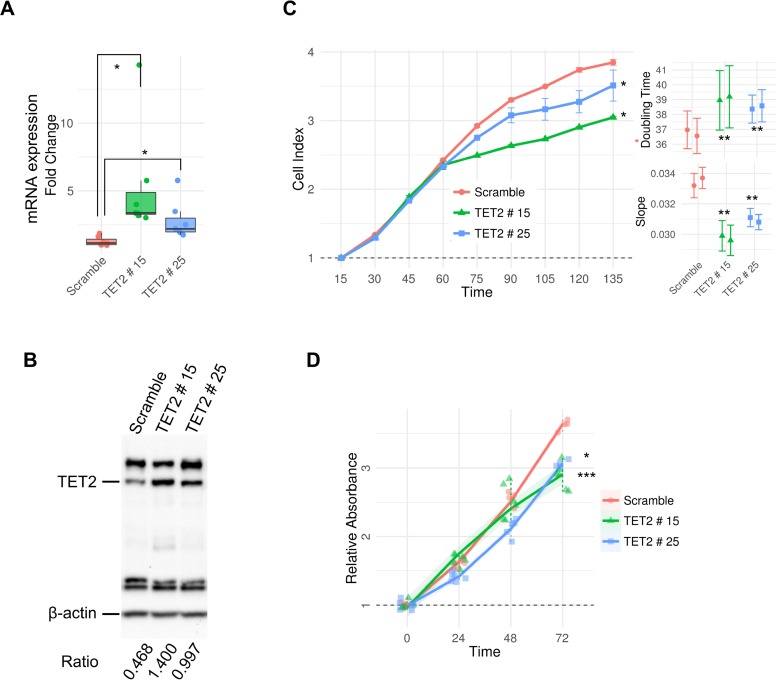
*In vitro* effects of TET2 restoration in LN229 glioblastoma cell line (**A**) qRT-PCR of TET2 transfected variant 1 in two clones of LN229. Expression levels were determined by real time-PCR and data, are expressed as a ratio with respect to GAPDH. Fold change was calculated relative to scramble. (**B**) WB analysis against TET2 in the same LN229 clones. Ratios between TET2 and β-actin intensity values calculated with ChemiDoc™ XRS^+^ software are shown. (**C**) Proliferation rate was represented as cell index units. The graphic shows average and standard deviation of two technical replicates of each clone over 135 hours. Slope (1/hours) and doubling time (hours), analyzed during the exponential growth phase (15–72 h), is also shown (right panels). (**D**) Cell viability was determined by MTT assay on scramble and TET2-stably transfected cells. The average absorbance in six replicates at various time points, normalized with respect to the average absorbance at time 0 h for each sample, are represented. Wilcoxon signed rank test and general linear models were applied and *p*-values were adjusted applying the Bonferroni correction. ^*^p-value < 0.05; ^***^p-value < 0.001.

We next wanted to determine whether TET2 inactivation blocked cell growth suppression in brain malignancies which displayed TET2 epigenetic-associated downregulation. To address this issue, we transfected TET2 in the glioblastoma cell line LN229, which exhibits hypo-hydroxymethylation as well as downregulation of TET2 mRNA (Figure 3). TET2 transfection resulted in the upregulation of TET2 mRNA (Wilcoxon rank sum test, *p*-value < 0.05) (Figure 3A) and protein levels (Figure 3B). Impedance-based cellular growth assays revealed that TET2-transfected cells (two clones) grew slightly less than the control cells (Wilcoxon rank sum test, *p*-value < 0.05 in both clones) (Figure 3C) as well as finding significant differences in population doubling time and slope between control and TET2-overexpressing clones (General linear models *p*-value < 0.01 in both clones) (Figure 3C). In addition, overexpression of TET2 resulted in a decrease in cell viability (General linear model, *p*-value < 0.05) (Figure 3D).

### TET2 expression is associated with glioblastoma growth *in vivo*

**Figure 4:**
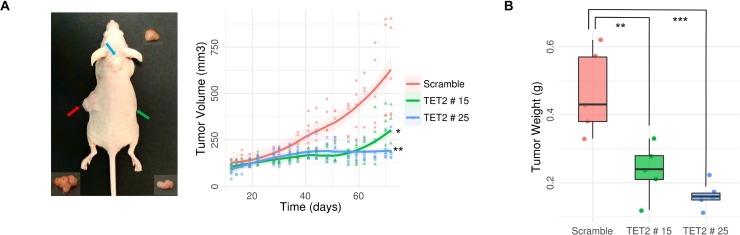
Antitumoral effects of TET2 restoration on tumor growth *in vivo* (**A**) Scramble (on left, represented in red) and TET2 transfected LN229 cells (TET2 # 15 on right, in green, and TET2 # 25 on neck base, in blue) were s.c. injected into nude mice (*n* = 5) (representative image on the left). Evolution of tumor growth was measured regularly for 72 days. Statistically significant differences in tumor growth (A) and weight (**B**) were shown at the endpoint. General linear models were applied, and *p*-values were adjusted applying the Bonferroni correction. ^*^p-value < 0.05; ^**^p-value < 0.01; ^***^p-value < 0.001.

To study the effect of TET2 expression in glioblastoma *in vivo* we tested the ability of TET2-transfected LN229 cells to form tumors in nude mice as compared with control cells transfected with an empty vector (Figure 4A). Negative control of LN229 glioblastoma cells resulted in tumors with exponential growth (Figure 4A). In contrast, two clones of LN229 cells which overexpress TET2 (Figure 3A, Supplementary Figure 4) had much lower tumorigenic potential (Figure 4A). Mice were sacrificed 72 days after tumor-xenograft implantation. At that time, tumors are around two times larger in mice xenografted with cells transfected with the empty vector than in mice transfected with cells overexpressing TET2 (Linear model, *p*-value < 0.05) (Figure 4A). Tumor weight was also notably different between the two clones and the scramble (Linear model, *p*-value < 0.005) (Figure 4B).

### TET2 regulates neural differentiation in glioblastoma cells

**Figure 5:**
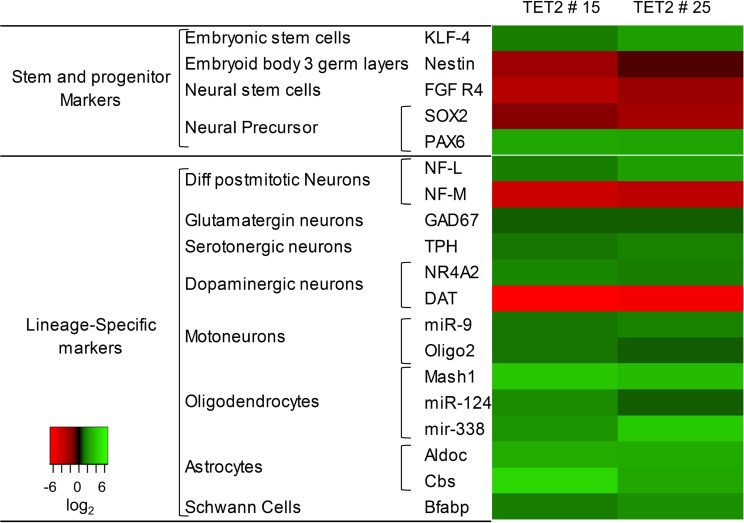
Regulation of neural-lineage markers is associated with TET2 restoration mRNA expression of 92 neural related genes was analyzed with the neural lineage qPCR Profiler Kit (System Biosciences). TET2 clones were compared with scramble. For both clones, the heatmap representation includes genes with at least 2-fold expression changes (overexpression log_2_ ≥ 1, green scale; downregulation log_2_ ≤ −1, red scale).

The effect of TET2 overexpression on glioblastoma cell growth and viability, together with previous reports suggesting that this enzyme plays an important role in neural differentiation [32–34] led us to postulate the hypothesis that the antitumoral effect of TET2 in brain tumors is mediated, at least in part, by its role in this process. To address this issue, we used a Neural Lineage Profiler assay to compare the relative expression of more than 92 markers of different stages of human neuroectodermal differentiation between control and TET2-transfected LN229 cells (Supplementary Table 1). Results showed that TET2 upregulation consistently altered the expression of 19 neuroectodermal markers in both TET2 clones (Figure 5, Supplementary Table 1). The greatest changes were observed for the Proneural transcriptor factor/Neural and Oligodendrocyte precursor Mash1 [35] and Cystathionine β-synthase (cbs), an enzyme with high expression levels in astrocyte lineages [36].

## DISCUSSION

The tet methylcytosine dioxygenase 2 (TET2), as well as its homologues TET1 and TET3, catalyze the conversion of the epigenetic chemical modification 5-methylcytosine (5 mC) into 5-hydroxymethylcytosine (5 hmC), which is subsequently oxidized and is converted into 5-formylcytosine (5fC) and 5-carboxylcytosine (5caC) [2]. This knowledge initially led to the notion that TET enzymes play an important role in active DNA demethylation [37, 38]. However, recent studies showing that 5 hmC might be an epigenetic mark in its own right suggest that the role of TET enzymes is not simply restricted to actively demethylating genomic DNA [3, 39].

An especially high expression of TET2 has been found in hematopoietic tissues [40] suggesting it has an important role to play in this process, which is supported by the fact that TET2 is frequently mutated in hematological malignancies [41–43].

Although at lower frequencies than observed in hematological malignancies, TET2 alterations have also been found in various types of solid tumors, such as renal cell carcinoma [44], metastatic castration-resistant prostate cancer (mCRPC) [45], lung [46], colon and rectal cancer [47]. However, these mutations cannot always explain the frequently altered expression of this protein in cancer [19, 48, 49]. Interestingly, although TET2 is not frequently mutated in glioblastoma [50–52], it has been found to be frequently downregulated [53].

To identify alternative molecular mechanisms involved in TET2 downregulation in glioblastoma we considered the possible role of epigenetic factors by analyzing the TET2 promoter and intergenic levels of 5 mC, 5 hmC and H4K16ac in control and tumoral samples. Our results demonstrated that there were alterations of all the epigenetic marks analyzed.

At a functional level, the restoration of TET2 activity in the LN229 glioblastoma cell line reduced cell proliferation *in vitro*, and tumor growth *in vivo*, which is in agreement with previous work suggesting that TET2 has antitumor activity in leukemias [21, 54] and in other types of tumors including parathyroid carcinoma [55], colorectal cancer [20] and glioma [53]. These results, then, suggest that TET2 activity is frequently disrupted in cancer through different molecular mechanisms and that this process might contribute to malignant transformation.

Our results show that restoration of TET2 expression in glioblastoma cells tends to upregulate genes involved in neural differentiation including the Brain fatty acid-binding protein (BFABP), implicated in glial lineage differentiation [56], the proneural basic helix–loop–helix (bHLH) transcription factor Mash1, related to oligodendrogenesis and neural precursor differentiation [57–61] and Cystathionine β-Synthase (cbs), whose protein expression is detected at high levels in astrocytes [62]. Interestingly, low expression of cbs was found in gliomas [63]. These results therefore suggest that TET2 might be involved in neural differentiation, which is in line with previously published data [32, 64, 65].

It has previously been shown that MicroRNAs (miRNAs) play an important role in neuronal development and neurogenesis [66, 67]. We also detected changes in neuron-specific microRNA miR-124 [66] and oligodendrocyte-specific microRNA miR-338 [67] expression when TET2 activity is restored in LN229 glioblastoma cell line. In contrast, neural progenitor markers of early stages of differentiation were slightly downregulated as Sox2 [68, 69]. These results are in agreement with previous data suggesting that TET2 plays an important role in neural development [17, 33, 70, 71] and support the notion that the antitumoral effect of TET2 in glioblastoma might be mediated, at least in part, through its role in the regulation of neuroectodermal differentiation.

## MATERIALS AND METHODS

### Human cell lines and human samples

The human glioblastoma cell lines (LN229, LN-18, T98-G and U-87 MG) were cultured according to American Type Culture Collection (ATCC) recommendations and authenticated using short tandem repeat profiling of an extracted DNA sample employing an AmpFℓSTR Identifiler for high resolution screening and intraspecies cross-contamination detection (Bio-Synthesis, Inc). Cell lines were cultured in our laboratory at 37°C in 5% CO_2_ in Dulbecco´s Modified Eagle´s Medium (DMEM) (Gibco, 41965) supplemented with 10% fetal bovine serum (FBS) (F6178, Sigma), 2% penicillin/streptomycin (Gibco, 15070) and 1% Amphotericin B (Gibco, 15290).

Healthy human biopsy (*n* = 5) and tumor samples (*n* = 11) were obtained from the Institute of Oncology of Asturias Tumor Bank (Asturias, Spain). Histopathological study had previously been confirmed at the Hospital Universitario Central de Asturias, and informed consent was obtained from all patients before sample collection. Samples were frozen at −80°C until further analysis. A second group of donated samples came from The Banc De Teixits Neurològics-Biobanc Hospital Clínic-IDIBAPS (Barcelona, Spain) and The Biobanco Hospital Virgen de la Salud (Toledo, Spain). Tissue collection and all analyses were approved by the appropriate institutional review boards in accordance with national and EU guidelines.

### DNA methylation analysis with high-density array

Microarray-based DNA methylation profiling was performed on 5 healthy samples and 9 tumoral samples using the HumanMethylation 450 BeadChip array [72]. Oxidative bisulfite (oxBS) and bisulfite-only (BS) conversion was performed using the TrueMethyl^®^ protocol for 450K analysis (Version 1.1, CEGX) following the manufacturer's recommended procedures. Processed DNA samples were then hybridized to the BeadChip (Illumina), following the Illumina Infinium HD Methylation Protocol. Genotyping services were provided by the Spanish Centro Nacional de Genotipado (CEGEN-ISCIII) (www.cegen.org).

Raw IDAT files were processed using the R/Bioconductor package minfi [73] (version 1.14.0), implementing the SWAN algorithm [74] to correct for differences in microarray probe design. Probes where at least two samples had detection *p*-values > 0.01, and samples where at least 5500 probes had detection *p*-values > 0.01 were filtered out. *M*-values and beta values were then computed as the final step in the preprocessing procedure. In line with a previously published methodology [75], *M*-values were used for the statistical analyses and beta values for effect size thresholding, visualization and report generation.

Beta values from oxBS samples were subtracted from their corresponding BS treated pairs, generating an artificial dataset representing the level of 5 hmC for each probe and sample as per a previously published methodology [76]. One further dataset, representing 5 mC levels, was created using the beta values from oxBS samples. Differential methylation and hydroxymethylation of an individual probe was determined by a moderated *t*-test implemented in the R/Bioconductor package limma [77].

### Bisulfite pyrosequencing

The DNA methylation and hydroxymethylation status of representative CpG sites were evaluated in 12 healthy and 22 tumoral samples, and in glioblastoma cell lines using bisulfite pyrosequencing. Genomic DNA was isolated following standard phenol-chloroform extraction protocols. Oxidative bisulfite (oxBS) and bisulfite-only (BS) conversion of DNA was performed following an adapted protocol from the TrueMethyl Array Kit User Guide (CEGX, Version 2). DNA samples were cleaned with Agencourt AMPure XP (Beckman Coulter) and then oxidized using KRuO4 (Alpha Aeser) solution (375 mM in 0.3 M NaOH). Finally, BS conversion was carried out using Epitect bisulfite Kit (Qiagen). Polymerase chain reaction (PCR) amplification of the regions of interest was performed using specific primers (see Supplementary Table 2) designed using the software PyroMark assay design (v. 2.0.01.15). Pyrosequencing was conducted using PyroMark Q24 reagents, vacuum prep Workstation, equipment, and software (Qiagen).

### 5-hydroxymethylcytosine immunoprecipitation-qPCR assay

Immunoprecipitation of 5 hmC was carried out using the EpiQuik Hydroxymethylated DNA Immunoprecipitation (hMeDIP) Kit (Epigentek), according to the manufacturer's instructions. Input, non-specific IgG- and 5 hmC-enriched fractions were obtained from ten samples corresponding to five normal brains and five glioma tumors. All fractions were amplified by qPCR with oligonucleotides specific to the CpG position detailed in Supplementary Table 2. After confirming there were no significant differences between input DNAs, 5 hmC relative enrichment was calculated as a Fold Change relative to Input Ct Mean.

### Chromatin immunoprecipitation assay

Chromatin immunoprecipitation was performed with control brain and glioma samples. The assay was performed as described in the protocol “Chromatin preparation from tissues for chromatin immunoprecipitation (ChIP)” by Abcam, with some minor modifications. Frozen tissue was fixed for 10 min with 1–1.5% formaldehyde solution. Crosslinking was halted by the addition of glycine to 125 mM. Homogenization was performed on ice using a Micropistille Eppendorf tightly fitted into the bottom of the eppendorf tube. Samples were resuspended in Lysis Buffer (50 mM HEPES-KOH pH7.5;140 mM NaCl; 1 mM EDTA pH8; 1% Triton X-100; 0.1% Sodium Deoxycholate; 0.1% SDS; Protease Inhibitors) and sonicated. Sonication was performed by Diagenode's Bioruptor^®^ Sonicator to obtain chromatin fragments of 200–500 bp in length. Immunoprecipitations were performed overnight using antibodies against H4K16ac (Active Motif, 39167), with total histone H3 (Abcam, ab1791) as positive control, and IgG antiserum (Abcam, ab46540) as negative control. Antibody–chromatin complexes were precipitated with Salmon Sperm DNA/Protein A-Agarose beads (Upstate Biotechnologies), then washed and eluted using the corresponding buffer (1% SDS,100mM NaHCO3). DNA was extracted with phenol–chloroform and was ethanol-precipitated. Immunoprecipitated DNA was analyzed by real-time PCR on TET2 promoter region (Supplementary Table 2) using SYBR^®^ Green in the StepOnePlus™ Real-Time PCR System (Applied Biosystems). All measurements were performed in triplicate, non-template controls were included, and a calibration curve was determined for each primer set.

### qRT-PCR

Total RNA was extracted from LN229 glioblastoma cell line and human samples using TRIzol Reagent (Life Technologies). Residual genomic DNA was removed with DNA-free kit (Invitrogen) and cDNA synthesis was performed using SuperScript III reverse transcriptase (Invitrogen) following the manufacturer´s instructions. Quantitative PCR reactions were tested in triplicate using SYBR Green PCR Master Mix (Applied Biosystems) and specific primers in StepOnePlus™ Real-Time PCR System (Applied Biosystems). Data were analyzed by the double delta Ct method, and gene expression was normalized to glyceraldehyde-3-phosphate-dehydrogenase (GAPDH) as endogenous control. All primers were acquired from Sigma. Oligonucleotide sequences are listed in Supplementary Table 2.

The cDNA used in the Neural Lineage qPCR Profiler Kit (System Biosciences) was synthesized according to the manufacturer´s instructions and quantitative PCR reactions were performed in HT7900 Real Time PCR System (Applied Biosystems).

### Immunohistochemistry

For the immunohistochemical analysis of protein levels we used the EnVision FLEX Mini Kit (DAKO, K8024) and Dako Autostainer system. Paraffin embedded tissues (3–5 μm) were deparaffinized, rehydrated, and then epitopes were retrieved by heat induction (HIER) at 95°C for 20 min at pH 6 (DAKO, GV805) in the Pre-Treatment Module, PT-LINK (DAKO).

Sections were incubated with TET2 antibody (Abcam, Ab94580) diluted in EnVision™ FLEX Antibody Diluent (DAKO, K8006) (1:500 dilution) for 30 min after blocking endogenous peroxidase with EnVision™ FLEX Peroxidase-Blocking Reagent (DAKO, DM821). The signal was detected using diaminobenzidine chromogen as substrate after incubation with Dako EnVision™ FLEX /HRP (DAKO, DM822). Sections were counterstained with hematoxylin. Appropriate negative controls were also tested.

After the complete process, sections were dehydrated and mounted with permanent medium (Dako mounting medium, CS703).

### TET2 transfection

LN229 cell line was transfected in order to overexpress TET2 (GeneCopedia, EX-H3630-M43), incorparating a non-effective Scramble construct as negative control (GeneCopedia, EX-EGFP-M43), using Lipofectamine 3000 transfection Kit (Invitrogen, L3000015) as per the manufacturer´s recommendations. The cells were collected 72 h after transfection and stable transfectants were obtained after selection with 1 mg/ml G418 disulfate salt (Sigma, A-1720). TET2 expression was checked by qRT-PCR using the specific primers for TET2 transcription variant1 detection (Supplementary Table 2). TET2 expression was confirmed in positive clones by Western blot (WB) using anti-TET2 antibody (1:500, ab1789, Abcam) and anti-β-actin (1:5000, sc-47778, Santa Cruz) as a loading control.

### Cell growth experiments

Cell proliferation rate was measured with the iCELLigence real-time cell analyzer (RTCA) (ACEA Biosciences). Duplicates of LN229 transfected cells (15 × 10^3^ cells) were seeded onto analyzer specific E-Plates L8. Cell impedance was measured by continuous monitoring every 3 hours for 6 days (135 h) through micro electric biosensors located at the base of the plate wells. Data were exported from the cell analyzer instrument to the control unit and RTCA software was used to analyze slope and doubling time parameters. As cell growth increases, more cells attach to the plate electrodes leading to an increase in the impedance measured by the instrument. Cell index was the parameter used to quantify the relative change in electrical impedance. Data were normalized after 15 hours.

### Cell viability assay

Cell viability was determined by 3-(4,5-Dimethylthiazol-2-yl)-2,5-Diphenyltetrazolium Bromide (MTT) assay on stably transfected cells, as described by Mosmann and colleagues [78]. Six replicates (2 × 10^3^ cells) per condition and time point were established in 96-well plates. MTT (500 μg/ml) (Sigma, M5655) was added to medium and kept at 37°C and 5% CO_2_. After 3 hours, the MTT was removed and the Formazan crystals formed were dissolved in dimethyl sulfoxide (DMSO) (100 μl/well) (Sigma, D5879-M) and absorbance at 595 nm was measured with an automated microtiter plate reader.

### Xenografts

Five-week old NU/NU female mice (Charles River Japan Inc. Kanagawa, Japan) were subcutaneously injected with 1 × 10^6^ cells mixed 1:1 with BD Matrigel Matrix High Concentration (BD Biosciences, Erembodegem, Belgium) which had been previously diluted 1:1 with culture medium. Both, TET2 and scramble transfected cells were injected into five nude mice. Tumor size was measured with calipers twice a week and tumor volume was determined as V = 4/3π(Rr)^2^ were R is the maximum diameter and r is the minimum diameter. Mean tumor volume was calculated for the corresponding group. After sacrifice, the tumors were excised and weighed.

### Statistical analysis

Group comparisons were performed by fitting a general linear model to each dataset. Contrasts were defined to test the different hypotheses (comparisons between levels of the main group variable) on each of the models. Repeated measures designs, involving the measure of a quantity across different time points, were modeled using a general linear model, with a main group predictor and a blocking variable describing the time of observation. Time was modeled as an ordinal variable. Contrasts were defined over the main group variable. Correlation between variables was assessed using the Spearman rank correlation coefficient.

The resulting *p*-values were adjusted using the Bonferroni method for controlling the Family-Wise Error Rate (FWER). Goodness-of-fit was assessed for each of the models using graphical methods and the Shapiro-Wilk test on the residuals. A Wilcoxon rank-sum (group comparisons) or signed-rank (comparisons across time) test was used for each pairwise comparison if the residuals showed strong deviations from normality and there were enough observations.

All statistical analyses were conducted using the R statistical programming language (version 3.4.0) (R Development Core Team 2008) [79].

## SUPPLEMENTARY MATERIALS FIGURES AND TABLES





## References

[1] Tahiliani M, Koh KP, Shen Y, Pastor WA, Bandukwala H, Brudno Y, Agarwal S, Iyer LM, Liu DR, Aravind L, Rao A (2009). Conversion of 5-methylcytosine to 5-hydroxymethylcytosine in mammalian DNA by MLL partner TET1. Science.

[2] Ito S, Shen L, Dai Q, Wu SC, Collins LB, Swenberg JA, He C, Zhang Y (2011). Tet proteins can convert 5-methylcytosine to 5-formylcytosine and 5-carboxylcytosine. Science.

[3] Lopez V, Fernandez AF, Fraga MF (2017). The role of 5-hydroxymethylcytosine in development, aging and age-related diseases. Ageing Res Rev.

[4] Ko M, An J, Bandukwala HS, Chavez L, Aijo T, Pastor WA, Segal MF, Li H, Koh KP, Lahdesmaki H, Hogan PG, Aravind L, Rao A (2013). Modulation of TET2 expression and 5-methylcytosine oxidation by the CXXC domain protein IDAX. Nature.

[5] Lyu Y, Lou J, Yang Y, Feng J, Hao Y, Huang S, Yin L, Xu J, Huang D, Ma B, Zou D, Wang Y, Zhang Y (2017). Dysfunction of the WT1-MEG3 signaling promotes AML leukemogenesis via p53-dependent and -independent pathways. Leukemia.

[6] Wang Y, Xiao M, Chen X, Chen L, Xu Y, Lv L, Wang P, Yang H, Ma S, Lin H, Jiao B, Ren R, Ye D (2015). WT1 recruits TET2 to regulate its target gene expression and suppress leukemia cell proliferation. Mol Cell.

[7] Song SJ, Ito K, Ala U, Kats L, Webster K, Sun SM, Jongen-Lavrencic M, Manova-Todorova K, Teruya-Feldstein J, Avigan DE, Delwel R, Pandolfi PP (2013). The oncogenic microRNA miR-22 targets the TET2 tumor suppressor to promote hematopoietic stem cell self-renewal and transformation. Cell Stem Cell.

[8] Guilhamon P, Eskandarpour M, Halai D, Wilson GA, Feber A, Teschendorff AE, Gomez V, Hergovich A, Tirabosco R, Fernanda Amary M, Baumhoer D, Jundt G, Ross MT (2013). Meta-analysis of IDH-mutant cancers identifies EBF1 as an interaction partner for TET2. Nat Commun.

[9] Dawlaty MM, Breiling A, Le T, Barrasa MI, Raddatz G, Gao Q, Powell BE, Cheng AW, Faull KF, Lyko F, Jaenisch R (2014). Loss of Tet enzymes compromises proper differentiation of embryonic stem cells. Dev Cell.

[10] Dawlaty MM, Breiling A, Le T, Raddatz G, Barrasa MI, Cheng AW, Gao Q, Powell BE, Li Z, Xu M, Faull KF, Lyko F, Jaenisch R (2013). Combined deficiency of Tet1 and Tet2 causes epigenetic abnormalities but is compatible with postnatal development. Dev Cell.

[11] Ko M, Bandukwala HS, An J, Lamperti ED, Thompson EC, Hastie R, Tsangaratou A, Rajewsky K, Koralov SB, Rao A (2011). Ten-Eleven-Translocation 2 (TET2) negatively regulates homeostasis and differentiation of hematopoietic stem cells in mice. Proc Natl Acad Sci U S A.

[12] Li Z, Cai X, Cai CL, Wang J, Zhang W, Petersen BE, Yang FC, Xu M (2011). Deletion of Tet2 in mice leads to dysregulated hematopoietic stem cells and subsequent development of myeloid malignancies. Blood.

[13] Langemeijer SM, Kuiper RP, Berends M, Knops R, Aslanyan MG, Massop M, Stevens-Linders E, van Hoogen P, van Kessel AG, Raymakers RA, Kamping EJ, Verhoef GE, Verburgh E (2009). Acquired mutations in TET2 are common in myelodysplastic syndromes. Nat Genet.

[14] Zhang W, Shao ZH, Fu R, Wang HQ, Li LJ, Wang J, Qu W, Liang Y, Wang GJ, Wang XM, Wu Y, Liu H, Song J (2012). TET2 Expression in Bone Marrow Mononuclear Cells of Patients with Myelodysplastic Syndromes and Its Clinical Significances. Cancer Biol Med.

[15] Li D, Guo B, Wu H, Tan L, Lu Q (2015). TET Family of Dioxygenases: Crucial Roles and Underlying Mechanisms. Cytogenet Genome Res.

[16] Rudenko A, Dawlaty MM, Seo J, Cheng AW, Meng J, Le T, Faull KF, Jaenisch R, Tsai LH (2013). Tet1 is critical for neuronal activity-regulated gene expression and memory extinction. Neuron.

[17] Santiago M, Antunes C, Guedes M, Sousa N, Marques CJ (2014). TET enzymes and DNA hydroxymethylation in neural development and function-how critical are they?. Genomics.

[18] Ficz G, Gribben JG (2014). Loss of 5-hydroxymethylcytosine in cancer: cause or consequence?. Genomics.

[19] Rasmussen KD, Helin K (2016). Role of TET enzymes in DNA methylation, development, and cancer. Genes Dev.

[20] Huang Y, Wang G, Liang Z, Yang Y, Cui L, Liu CY (2016). Loss of nuclear localization of TET2 in colorectal cancer. Clin Epigenetics.

[21] Mercher T, Quivoron C, Couronne L, Bastard C, Vainchenker W, Bernard OA (2012). TET2, a tumor suppressor in hematological disorders. Biochim Biophys Acta.

[22] Delhommeau F, Dupont S, Della Valle V, James C, Trannoy S, Masse A, Kosmider O, Le Couedic JP, Robert F, Alberdi A, Lecluse Y, Plo I, Dreyfus FJ (2009). Mutation in TET2 in myeloid cancers. N Engl J Med.

[23] Pan F, Weeks O, Yang FC, Xu M (2015). The TET2 interactors and their links to hematological malignancies. IUBMB Life.

[24] Medeiros BC, Fathi AT, DiNardo CD, Pollyea DA, Chan SM, Swords R (2017). Isocitrate dehydrogenase mutations in myeloid malignancies. Leukemia.

[25] Lian CG, Xu Y, Ceol C, Wu F, Larson A, Dresser K, Xu W, Tan L, Hu Y, Zhan Q, Lee CW, Hu D, Lian BQ (2012). Loss of 5-hydroxymethylcytosine is an epigenetic hallmark of melanoma. Cell.

[26] Murata A, Baba Y, Ishimoto T, Miyake K, Kosumi K, Harada K, Kurashige J, Iwagami S, Sakamoto Y, Miyamoto Y, Yoshida N, Yamamoto M, Oda S (2015). TET family proteins and 5-hydroxymethylcytosine in esophageal squamous cell carcinoma. Oncotarget.

[27] Song F, Amos CI, Lee JE, Lian CG, Fang S, Liu H, MacGregor S, Iles MM, Law MH, Lindeman NI, Montgomery GW, Duffy DL, Cust AE (2014). Identification of a melanoma susceptibility locus and somatic mutation in TET2. Carcinogenesis.

[28] Abdel-Wahab O, Mullally A, Hedvat C, Garcia-Manero G, Patel J, Wadleigh M, Malinge S, Yao J, Kilpivaara O, Bhat R, Huberman K, Thomas S, Dolgalev I (2009). Genetic characterization of TET1, TET2, and TET3 alterations in myeloid malignancies. Blood.

[29] Deng W, Wang J, Zhang J, Cai J, Bai Z, Zhang Z (2016). TET2 regulates LncRNA-ANRIL expression and inhibits the growth of human gastric cancer cells. IUBMB Life.

[30] Nickerson ML, Das S, Im KM, Turan S, Berndt SI, Li H, Lou H, Brodie SA, Billaud JN, Zhang T, Bouk AJ, Butcher D, Wang Z (2017). TET2 binds the androgen receptor and loss is associated with prostate cancer. Oncogene.

[31] Taylor GC, Eskeland R, Hekimoglu-Balkan B, Pradeepa MM, Bickmore WA (2013). H4K16 acetylation marks active genes and enhancers of embryonic stem cells, but does not alter chromatin compaction. Genome Res.

[32] Hahn MA, Qiu R, Wu X, Li AX, Zhang H, Wang J, Jui J, Jin SG, Jiang Y, Pfeifer GP, Lu Q (2013). Dynamics of 5-hydroxymethylcytosine and chromatin marks in Mammalian neurogenesis. Cell Reports.

[33] Qiao Y, Wang X, Wang R, Li Y, Yu F, Yang X, Song L, Xu G, Chin YE, Jing N (2015). AF9 promotes hESC neural differentiation through recruiting TET2 to neurodevelopmental gene loci for methylcytosine hydroxylation. Cell Discov.

[34] Zhao X, Dai J, Ma Y, Mi Y, Cui D, Ju G, Macklin WB, Jin W (2014). Dynamics of ten-eleven translocation hydroxylase family proteins and 5-hydroxymethylcytosine in oligodendrocyte differentiation. Glia.

[35] Parras CM, Galli R, Britz O, Soares S, Galichet C, Battiste J, Johnson JE, Nakafuku M, Vescovi A, Guillemot F (2004). Mash1 specifies neurons and oligodendrocytes in the postnatal brain. EMBO J.

[36] Enokido Y, Suzuki E, Iwasawa K, Namekata K, Okazawa H, Kimura H (2005). Cystathionine beta-synthase, a key enzyme for homocysteine metabolism, is preferentially expressed in the radial glia/astrocyte lineage of developing mouse CNS. FASEB J.

[37] Kohli RM, Zhang Y (2013). TET enzymes, TDG and the dynamics of DNA demethylation. Nature.

[38] Wu SC, Zhang Y (2010). Active DNA demethylation: many roads lead to Rome. Nat Rev Mol Cell Biol.

[39] Dahl C, Gronbaek K, Guldberg P (2011). Advances in DNA methylation: 5-hydroxymethylcytosine revisited. Clin Chim Acta.

[40] Solary E, Bernard OA, Tefferi A, Fuks F, Vainchenker W (2014). The Ten-Eleven Translocation-2 (TET2) gene in hematopoiesis and hematopoietic diseases. Leukemia.

[41] Bernard OA, Delhommeau F, Fontenay M, Vainchenker W (2009). Mutations in TET2 in myeloid cancers [Article in French]. Med Sci (Paris).

[42] Langemeijer SM, Jansen JH, Hooijer J, van Hoogen P, Stevens-Linders E, Massop M, Waanders E, van Reijmersdal SV, Stevens-Kroef MJ, Zwaan CM, van den Heuvel-Eibrink MM, Sonneveld E, Hoogerbrugge PM (2011). TET2 mutations in childhood leukemia. Leukemia.

[43] Mullighan CG (2009). TET2 mutations in myelodysplasia and myeloid malignancies. Nat Genet.

[44] Sato Y, Yoshizato T, Shiraishi Y, Maekawa S, Okuno Y, Kamura T, Shimamura T, Sato-Otsubo A, Nagae G, Suzuki H, Nagata Y, Yoshida K, Kon A (2013). Integrated molecular analysis of clear-cell renal cell carcinoma. Nat Genet.

[45] Nickerson ML, Im KM, Misner KJ, Tan W, Lou H, Gold B, Wells DW, Bravo HC, Fredrikson KM, Harkins TT, Milos P, Zbar B, Linehan WM (2013). Somatic alterations contributing to metastasis of a castration-resistant prostate cancer. Hum Mutat.

[46] Jin Y, Shao Y, Shi X, Lou G, Zhang Y, Wu X, Tong X, Yu X (2016). Mutational profiling of non-small-cell lung cancer patients resistant to first-generation EGFR tyrosine kinase inhibitors using next generation sequencing. Oncotarget.

[47] Cancer Genome Atlas Network (2012). Comprehensive molecular characterization of human colon and rectal cancer. Nature.

[48] Scourzic L, Mouly E, Bernard OA (2015). TET proteins and the control of cytosine demethylation in cancer. Genome Med.

[49] Yang H, Liu Y, Bai F, Zhang JY, Ma SH, Liu J, Xu ZD, Zhu HG, Ling ZQ, Ye D, Guan KL, Xiong Y (2013). Tumor development is associated with decrease of TET gene expression and 5-methylcytosine hydroxylation. Oncogene.

[50] Kim YH, Pierscianek D, Mittelbronn M, Vital A, Mariani L, Hasselblatt M, Ohgaki H (2011). TET2 promoter methylation in low-grade diffuse gliomas lacking IDH1/2 mutations. J Clin Pathol.

[51] Kraus TF, Greiner A, Steinmaurer M, Dietinger V, Guibourt V, Kretzschmar HA (2015). Genetic Characterization of Ten-Eleven-Translocation Methylcytosine Dioxygenase Alterations in Human Glioma. J Cancer.

[52] Cancer Genome Atlas Research Network (2008). Comprehensive genomic characterization defines human glioblastoma genes and core pathways. Nature.

[53] Chen B, Lei Y, Wang H, Dang Y, Fang P, Wang J, Yang J, Liu L (2017). Repression of the expression of TET2 by ZEB1 contributes to invasion and growth in glioma cells. Mol Med Rep.

[54] Rasmussen KD, Jia G, Johansen JV, Pedersen MT, Rapin N, Bagger FO, Porse BT, Bernard OA, Christensen J, Helin K (2015). Loss of TET2 in hematopoietic cells leads to DNA hypermethylation of active enhancers and induction of leukemogenesis. Genes Dev.

[55] Barazeghi E, Gill AJ, Sidhu S, Norlen O, Dina R, Palazzo FF, Hellman P, Stalberg P, Westin G (2017). A role for TET2 in parathyroid carcinoma. Endocr Relat Cancer.

[56] Kurtz A, Zimmer A, Schnutgen F, Bruning G, Spener F, Muller T (1994). The expression pattern of a novel gene encoding brain-fatty acid binding protein correlates with neuronal and glial cell development. Development.

[57] Dromard C, Bartolami S, Deleyrolle L, Takebayashi H, Ripoll C, Simonneau L, Prome S, Puech S, Tran VB, Duperray C, Valmier J, Privat A, Hugnot JP (2007). NG2 and Olig2 expression provides evidence for phenotypic deregulation of cultured central nervous system and peripheral nervous system neural precursor cells. Stem Cells.

[58] Nakatani H, Martin E, Hassani H, Clavairoly A, Maire CL, Viadieu A, Kerninon C, Delmasure A, Frah M, Weber M, Nakafuku M, Zalc B, Thomas JL (2013). Ascl1/Mash1 promotes brain oligodendrogenesis during myelination and remyelination. J Neurosci.

[59] Parras CM, Hunt C, Sugimori M, Nakafuku M, Rowitch D, Guillemot F (2007). The proneural gene Mash1 specifies an early population of telencephalic oligodendrocytes. J Neurosci.

[60] Sommer L, Shah N, Rao M, Anderson DJ (1995). The cellular function of MASH1 in autonomic neurogenesis. Neuron.

[61] Yi SH, Jo AY, Park CH, Koh HC, Park RH, Suh-Kim H, Shin I, Lee YS, Kim J, Lee SH (2008). Mash1 and neurogenin 2 enhance survival and differentiation of neural precursor cells after transplantation to rat brains via distinct modes of action. Mol Ther.

[62] Lee M, Schwab C, Yu S, McGeer E, McGeer PL (2009). Astrocytes produce the antiinflammatory and neuroprotective agent hydrogen sulfide. Neurobiol Aging.

[63] Takano N, Sarfraz Y, Gilkes DM, Chaturvedi P, Xiang L, Suematsu M, Zagzag D, Semenza GL (2014). Decreased expression of cystathionine beta-synthase promotes glioma tumorigenesis. Mol Cancer Res.

[64] Li X, Yao B, Chen L, Kang Y, Li Y, Cheng Y, Li L, Lin L, Wang Z, Wang M, Pan F, Dai Q, Zhang W (2017). Ten-eleven translocation 2 interacts with forkhead box O3 and regulates adult neurogenesis. Nat Commun.

[65] Orr BA, Haffner MC, Nelson WG, Yegnasubramanian S, Eberhart CG (2012). Decreased 5-hydroxymethylcytosine is associated with neural progenitor phenotype in normal brain and shorter survival in malignant glioma. PLoS One.

[66] Makeyev EV, Zhang J, Carrasco MA, Maniatis T (2007). The MicroRNA miR-124 promotes neuronal differentiation by triggering brain-specific alternative pre-mRNA splicing. Mol Cell.

[67] Zhao X, He X, Han X, Yu Y, Ye F, Chen Y, Hoang T, Xu X, Mi QS, Xin M, Wang F, Appel B, Lu QR (2010). MicroRNA-mediated control of oligodendrocyte differentiation. Neuron.

[68] Hutton SR, Pevny LH (2011). SOX2 expression levels distinguish between neural progenitor populations of the developing dorsal telencephalon. Dev Biol.

[69] Zhang S, Cui W (2014). Sox2, a key factor in the regulation of pluripotency and neural differentiation. World J Stem Cells.

[70] Mi Y, Gao X, Dai J, Ma Y, Xu L, Jin W (2015). A Novel Function of TET2 in CNS: Sustaining Neuronal Survival. Int J Mol Sci.

[71] Wen L, Tang F (2014). Genomic distribution and possible functions of DNA hydroxymethylation in the brain. Genomics.

[72] Bibikova M, Barnes B, Tsan C, Ho V, Klotzle B, Le JM, Delano D, Zhang L, Schroth GP, Gunderson KL, Fan JB, Shen R (2011). High density DNA methylation array with single CpG site resolution. Genomics.

[73] Fortin JP, Labbe A, Lemire M, Zanke BW, Hudson TJ, Fertig EJ, Greenwood CM, Hansen KD (2014). Functional normalization of 450k methylation array data improves replication in large cancer studies. Genome Biol.

[74] Maksimovic J, Gordon L, Oshlack A (2012). SWAN: Subset-quantile within array normalization for illumina infinium HumanMethylation450 BeadChips. Genome Biol.

[75] Du P, Zhang X, Huang CC, Jafari N, Kibbe WA, Hou L, Lin SM (2010). Comparison of Beta-value and M-value methods for quantifying methylation levels by microarray analysis. BMC Bioinformatics.

[76] Stewart SK, Morris TJ, Guilhamon P, Bulstrode H, Bachman M, Balasubramanian S, Beck S (2015). oxBS-450K: a method for analysing hydroxymethylation using 450K BeadChips. Methods.

[77] Ritchie ME, Phipson B, Wu D, Hu Y, Law CW, Shi W, Smyth GK (2015). Limma powers differential expression analyses for RNA-sequencing and microarray studies. Nucleic Acids Res.

[78] Mosmann T (1983). Rapid colorimetric assay for cellular growth and survival: application to proliferation and cytotoxicity assays. J Immunol Methods.

[79] Development Core Team R (2008). R: A Language and Environment for Statistical Computing. http://www.R-project.org.

