# Supplementary material for: Epigenetic dysregulation of TET2 in human glioblastoma
Source: Oncotarget. 2018 May 25;9(40):25922–34. doi: 10.18632/oncotarget.25406 (PMC5995234; doi:10.18632/oncotarget.25406)
Supplement: Supplementary file 1 [file oncotarget-09-25922-s001.pdf]

Epigenetic dysregulation of *TET2* in human glioblastoma

SUPPLEMENTARY MATERIALS

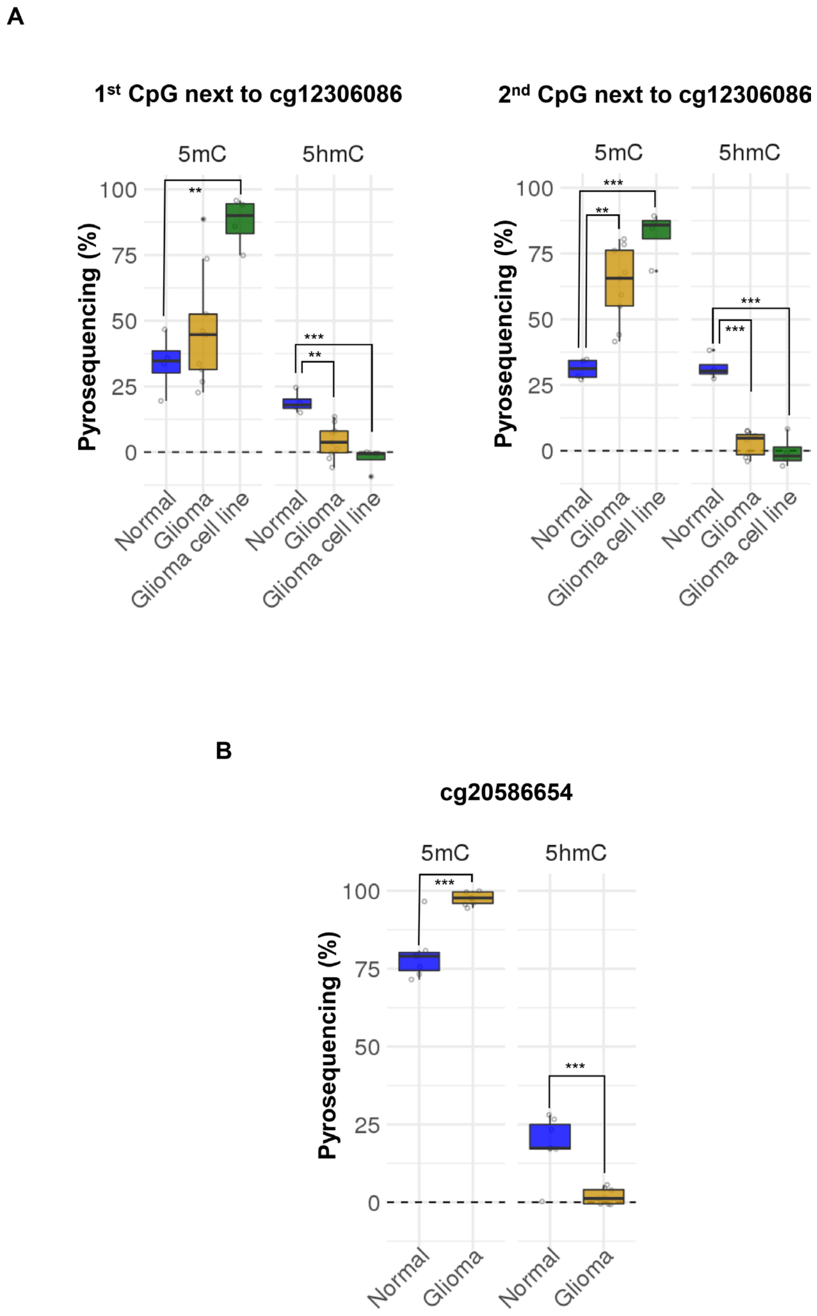

Supplementary Figure 1: Locus specific alterations of methylation and hydroxymethylation in glioma. (**A**) Box plot showing differences between the average percentage of 5mC and 5hmC (bisulfite pyrosequencing) at two neighboring CpG sites of cg12306086. Normal samples ($n = 4$), primary tumors ($n = 9$) and glioblastoma cell lines ($n = 4$) were analyzed. (**B**) Biological validation by pyrosequencing analysis of intragenic 5 mC and 5 hmC levels comparing 7 normal and 8 primary tumor samples. The averages for changes in percentage of methylation and hydroxymethylation between both groups is represented in a box plot for the CpG cg20586654. General linear models were applied and pvalues were adjusted by applying the Bonferroni correction. $^{**}p$-value < 0.01; $^{***}p$-value < 0.001.

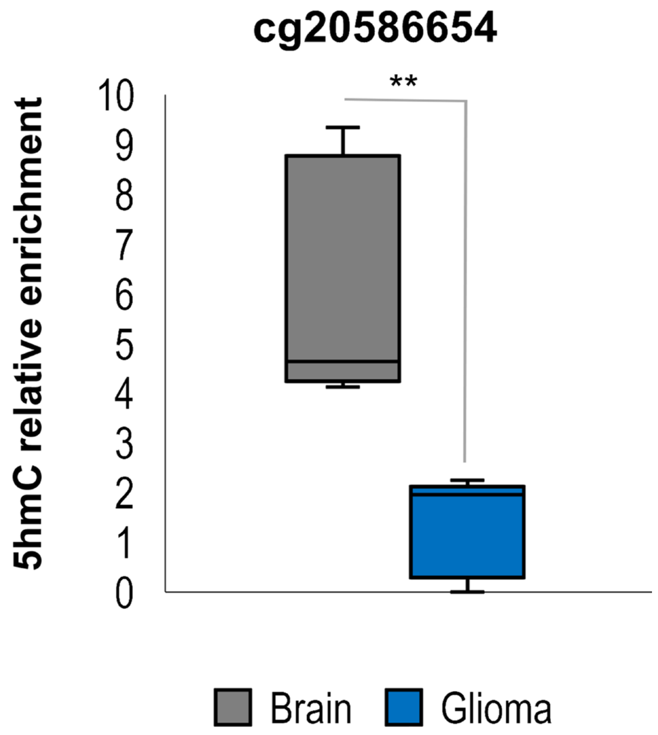

Supplementary Figure 2: Further validation of 5 hmC loss in glioma by immunoprecipitation and qRT-PCR at the cg20586654 CpG position. 5 brain samples and 5 gliomas were analyzed. $^{**}p < 0.01$.

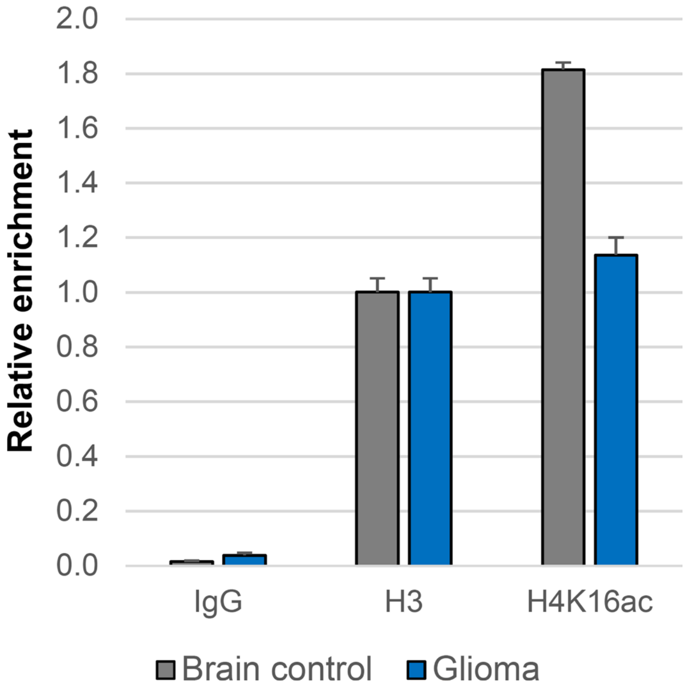

Supplementary Figure 3: H4K16ac relative fold enrichment at the *TET2* promoter region analyzed by chromatin immunoprecipitation (ChIP) in brain (gray bar) and glioma (blue bar) samples. Antibodies targeted at H3 (positive control) and IgG (negative control) were also used. A quantitative PCR analyses of each sample was performed by triplicate. Relative enrichments were calculated using the ΔΔCt method, and data were normalized firstly against IgG and then relativized against H3.

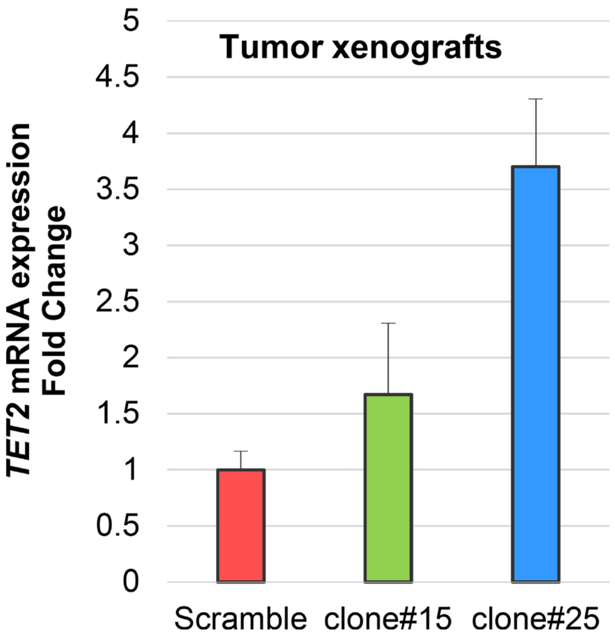

Supplementary Figure 4: qRT-PCR analysis of mRNA expression of *TET2* in tumor xenografts established from two clones of LN229 cells which overexpressed *TET2* (clone#15 and clone#25) compared with LN229 control cells transfected with an empty vector (Scramble). Data are expressed as a ratio in relation to *GAPDH*. Fold change was calculated relative to the scramble.

Supplementary Table 1: Complete list of data expression (Log2) obtained from 92 neural markers in TET2 transfected clones compared with scramble. Several genes related with stem cells, neural precursors and specific-neural lineage are included in the analysis. See Supplementary_Table_1

Supplementary Table 2: Primers used in this study. **Fw: forward; Rev: reverse; Seq: sequencing primer; [Btn]: biotin**

Pyrosequencing	
**Name**	**Sequence (5′→3′)**
TET2_CG12306086	Fw: TGTGAATTTAGGAATTTGAAGAATTTAGTA
	Rev: [Btn]ATACAACCTTTACCCTAAAAATTTCA
	Seq: ATTATATTGTTTTTTTAGATTTGTA
TET2_CG20586654	Fw: TTGTAAGGTGGAGGTGAATT
	Rev: [Btn]CCCCAAACAAACATACATCT
	Seq: GGTGGAGGTGAATTTTTAA
hMeDIP-qPCR	
**Name**	**Sequence (5′→3′)**
5hmC_TET2_cg120586654	Fw: GGCTGTAAGGTGGAGGTGAA
	Rev: TGCATCTTCTGCCTCTATGC
ChIP-qRT-PCR	
**Name**	**Sequence (5′→3′)**
TET2_promoter	Fw: TGGGCTTTGTTCTTCATCTCA
	Rev: GCTCTTCCTTTGACCGGAGT
qRT-PCR	
**Name**	**Sequence (5′→3′)**
TET2	Fw: ACGCTTGGAAGCAGGAGAT
	Rev: AAGGCTGCCCTCTAGTTGAA
TET2_Variant 1	Fw: AAAGATGAAGGTCCTTTTTATACCC
	Rev: ATAGCTTTACCCTTCTGTCCAAAC
GAPDH	Fw: AGCCACATCGCTCAGACAC
	Rev: CTCCATGGTGGTGAAGACG
