# Supplementary material for: Epigenetic dysregulation of TET2 in human glioblastoma
Source: Oncotarget. 2018 May 25;9(40):25922–34. doi: 10.18632/oncotarget.25406 (PMC5995234; doi:10.18632/oncotarget.25406)
Supplement: Supplementary file 2 [file oncotarget-09-25922-s002.docx]

**Supplementary Table 1: Complete list of data expression (Log2) obtained from 92 neural markers in TET2 transfected clones compared with scramble**

|  |  | TET2 # 15 | TET2 # 25 |
| --- | --- | --- | --- |
|  | **Gene** | **Log2** | **Log2** |
| **Embryonic stem cells** | Oct3/4 | -0.246387 | N/A |
|  | Nanog | N/A | N/A |
|  | KLF-4 | 1.386485 | 2.290385 |
|  | C-MYC | 0.502238 | 3.31024 |
| **Embryoid body 3 germ layers** | Nestin | -2.301213 | -0.833606 |
|  | Nodal | N/A | N/A |
|  | Brachyury (TBX1) | N/A | N/A |
|  | GSC | 6.788174 | 0.451 |
|  | AFP | N/A | N/A |
| **Neural stem cells** | SSEA-1 | 0.766991 | -0.334745 |
|  | CD133 | 0.814189 | 0.468206 |
|  | CXCR4 | N/A | N/A |
|  | FGF R4 | -3.170038 | -2.255293 |
| **Neural Precursor** | SOX1 | N/A | N/A |
|  | ROR2 | N/A | N/A |
|  | SOX2 | -1.641647 | -2.480265 |
|  | miR-128 | 0.274527 | 0.036274 |
|  | Activin A | 2.659447 | -0.103657 |
|  | PAX6 | 2.611317 | 2.400532 |
|  | TBR2 | -0.03275 | 0.00945 |
|  | NCAM | 0.161142 | 0.441738 |
|  | RMST | N/A | N/A |
|  | lncRNA-N1 | N/A | N/A |
|  | lncRNA-N2 | 3.248903 | -0.325205 |
| **Neuron-restricted progenitors** | MAP2 | 0.162333 | -0.150566 |
|  | SNCA synuclein, alpha | N/A | N/A |
|  | Doublecortin (DCX) | -0.787682 | 0.214151 |
| **Differentiated postmitotic Neurons** | NeuN | N/A | N/A |
|  | NF-L | 1.413261 | 2.385193 |
|  | NF-M | -4.084258 | -3.420941 |
|  | Synaptophysin | 0.309565 | -1.62136 |
|  | VAMP | -0.17667 | -0.155186 |
| **GABAergic neurons** | DARPP-32 (PPP1R1B) | 2.55397 | 0.237326 |
|  | EVF2 -LNC RNA | 1.193654 | -0.262 |
|  | Ptf1a | N/A | N/A |
|  | SLC32A1 | N/A | N/A |
| **Glutamatergin neurons** | GAD65 | N/A | N/A |
|  | GAD67 | 1.0004 | 0.996546 |
|  | SLC17A6 | N/A | N/A |
|  | SLC1A2 | N/A | 0.072098 |
|  | SLC1A3 | N/A | N/A |
| **Serotonergic neurons** | SLC6A4 | N/A | N/A |
|  | Tryptophan Hydroxylase | 1.22813 | 1.582279 |
|  | PET-1 | N/A | 2.634624 |
| **Cholinergic neurons** | VAChT (SLC18A3) | N/A | N/A |
| **Dopaminergic neurons** | TH | N/A | N/A |
|  | NR4A2 | 1.617976 | 1.451876 |
|  | KCNJ6 | -13.60127 | 0.87679 |
|  | FOXA2 | N/A | N/A |
|  | miR-133b | 0.627899 | 0.806247 |
|  | DAT | -6.664166 | -6.094051 |
| **Motoneurons** | HB9 | N/A | N/A |
|  | HOXC8 | 0.490788 | -0.545305 |
|  | miR-9 | 1.312715 | 1.580155 |
|  | Neurogenin-2 | 0.292 | 0.527738 |
|  | Oligo2 | 1.312836 | 0.94525 |
| **Peripheral Neurons** | PMP22 | 0.669819 | -0.26386 |
|  | PGP9.5 | -1.173512 | 0.458591 |
|  | Calretinin | N/A | N/A |
|  | HSP105 | -1.383394 | 0.497785 |
| **Glia restricted progenitors** | FGF Receptors | N/A | N/A |
|  | MAG | N/A | N/A |
| **Oligodendrocytes** | OSP | 5.038194 | -0.367279 |
|  | O4 | 0.215161 | -0.194704 |
|  | GalC | -0.988041 | 0.902092 |
|  | O1 | 0.891142 | 0.749454 |
|  | Sox10 | 0.910314 | 0.471462 |
|  | Mash1 | 3.809963 | 3.462834 |
|  | miR-124 | 1.773342 | 1.054898 |
|  | NSE | -0.464765 | 0.32472 |
|  | miR 219 | N/A | -1.144947 |
|  | mir-338 | 2.032053 | 3.868359 |
|  | Sox8 | 6.876907 | N/A |
|  | ABCA2 | 0.982506 | 0.263302 |
| **Astrocytes** | ALDH1L1 | N/A | N/A |
|  | GFAP | N/A | N/A |
|  | S100B | 1.790023 | -0.036904 |
|  | CD44 | -0.218257 | 0.28234 |
|  | Aldoc | 2.810537 | 2.783427 |
|  | Kcnj10 | 0.376797 | 1.625908 |
|  | Cbs | 4.596054 | 2.577841 |
|  | Sparcl1 | N/A | N/A |
| **Microglia** | CD11b | -0.331846 | -2.591886 |
|  | CD40 | -5.777418 | 3.989848 |
|  | CD68 | 0.168705 | -0.356667 |
|  | CD45 | N/A | N/A |
| **Schwann Cells** | Vimentin | -0.305372 | -0.610775 |
|  | MBP | N/A | N/A |
|  | PLP | -0.217335 | 0.945608 |
|  | Bfabp | 1.429388 | 1.868439 |
|  | miR-138 | 0.742512 | 0.866778 |
|  | 2', 3'-cyclic nucleotide 3'-phosphodiesterase (CNPase) | 1.052972 | 0.940168 |
| **Control** | GAPDH | -1.441729 | -1.833714 |
|  | Actin | -1.264969 | -0.433232 |
|  | Alu Sq | N/A | N/A |
|  | NTC | N/A | N/A |

Several genes related with stem cells, neural precursors and specific-neural lineage are included in the analysis.
